# Supplementary material for: Comparative analysis of high- and low-level deep learning approaches in microsatellite instability prediction
Source: Sci Rep. 2022 Jul 18;12:12218. doi: 10.1038/s41598-022-16283-3 (PMC9293930; doi:10.1038/s41598-022-16283-3)
Supplement: Supplementary file 1 — Supplementary Information. [file 41598_2022_16283_MOESM1_ESM.pdf]

Supplementary Information

Comparative analysis of high- and low-level deep learning approaches in microsatellite instability prediction

Jeonghyuk Park[1,*,+], Yul Ri Chung[2,*], and Akinao Nose[1,3]
[1]Department of Physics, Graduate School of Science, The University of Tokyo, Tokyo, Japan
[2]Pathology Center, Seegene Medical Foundation, Seoul, Korea
[3]Department of Complexity Science and Engineering, Graduate School of Frontier Sciences, The University of Tokyo, Chiba, Japan

Supplementary Figure 1-3

Supplementary Table 1-6

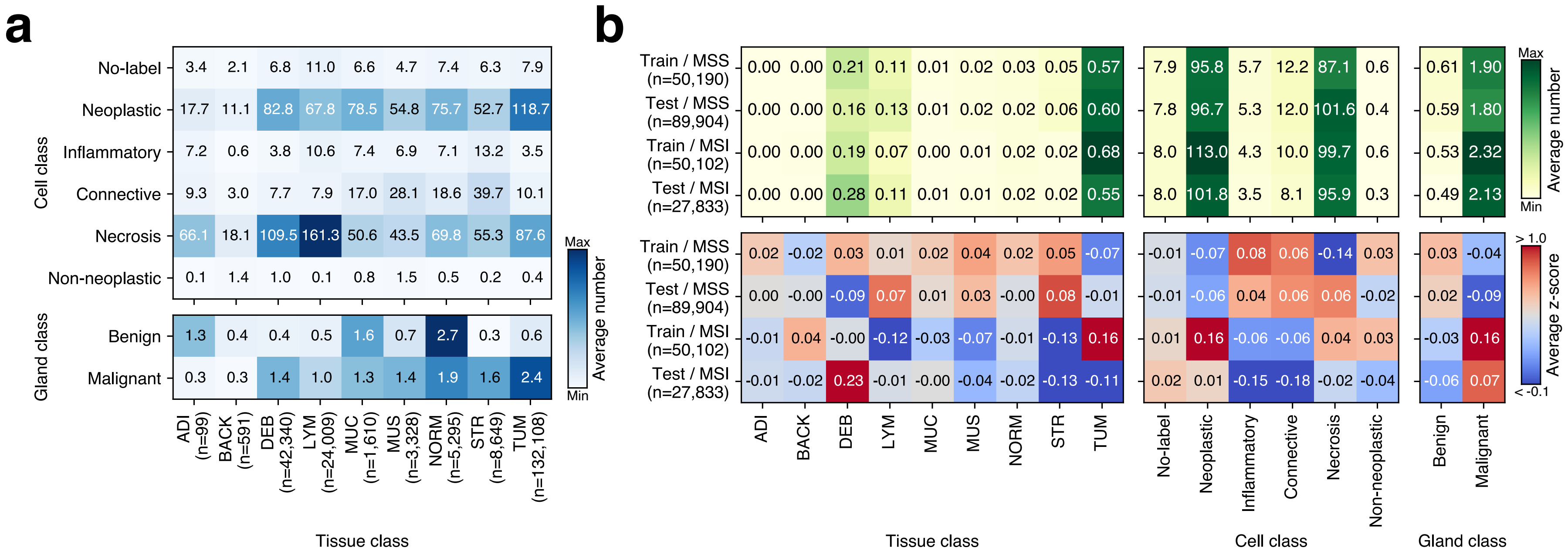

Supplementary Figure 1. (a) Correlations among low-level features derived from both the training and test sets from the stomach (STAD) cohort. (b) Low-level features of the training and test sets of the STAD cohort. Average number (top) and z score (bottom). ADI, adipose tissue; BACK, background; DEB, debris; LYM, lymphocytes; MUC, mucus; MUS, smooth muscle; NORM, normal colon mucosa; STR, cancer-associated stroma; TUM, colorectal adenocarcinoma epithelium. The heatmaps were created with Python v3.8.8 (https://www.python.org) and Matplotlib v3.4.3 (https://www.matplotlib.org). The figure was generated using Inkscape v1.1 (https://inkscape.org).

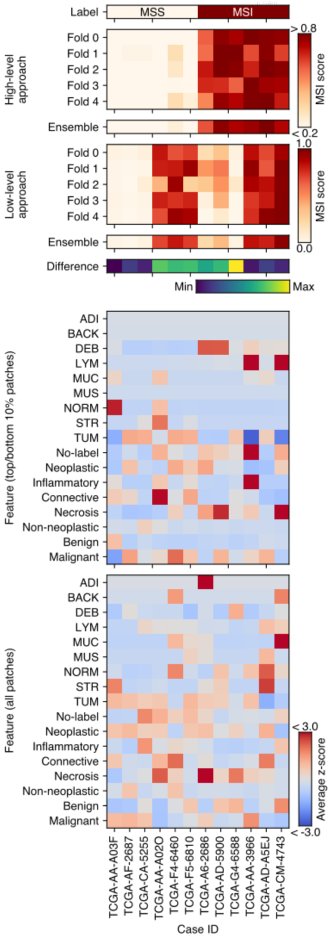

Supplementary Figure 2. Comparison of high/low-level approaches for select cases. Case-level prediction of EfficientNet (high-level approach) and LGBM (low-level approach) with MSI labels (top six panels). The predicted MSI scores of fivefold models and their ensemble are shown. Their difference is calculated based on the z scores of each ensemble. Representative image features derived from high/low-level approaches (bottom two panels). The average z score of the number of tissues, cells, and glands of the top 10\% of MSS/MSI patches and the case-level average. TCGA case IDs are shown as column labels. LGBM, light gradient boosting machine; MSS, microsatellite stable; MSI, microsatellite instable (instability); TCGA, the cancer genome atlas; ADI, adipose tissue; BACK, background; DEB, debris; LYM, lymphocytes; MUC, mucus; MUS, smooth muscle; NORM, normal colon mucosa; STR, cancer-associated stroma; TUM, colorectal adenocarcinoma epithelium. The heatmaps were created with Python v3.8.8 (https://www.python.org) and Matplotlib v3.4.3 (https://www.matplotlib.org). The figure was generated using Inkscape v1.1 (https://inkscape.org).

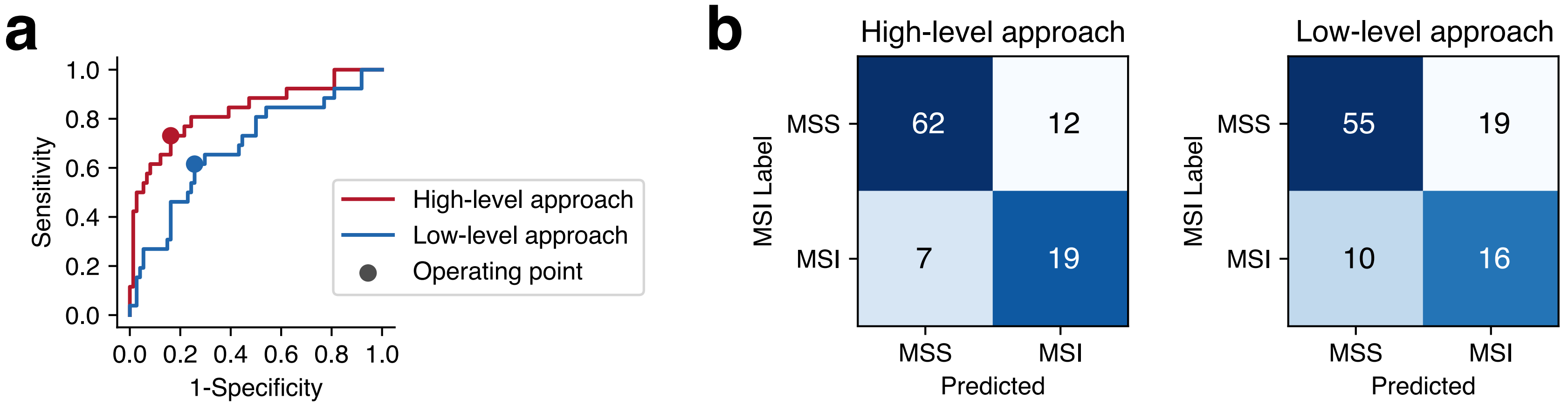

c

Approach	AUROC	Threshold (operating point)	Accuracy	Balanced accuracy	Sensitivity (Recall)	Specificity	Precision	F1-score
High-level (ensembled)	0.8316	0.40	0.8100	0.7843	0.7308	0.8378	0.6129	0.6667
Low-level (ensembled)	0.6866	0.35	0.7100	0.6793	0.6154	0.7432	0.4571	0.5246

Supplementary Figure 3. Classification performance of high-/low-level approaches. (a) Receiver operating characteristic curves of the high-level approach (red) and low-level approach (blue) on the test set. For each approach, we used the probabilities of the ensemble of fivefold models. The operating point was dete rmined as the threshold at the point where the F1-score of the test set was the maximum, while the threshold was adjusted from 0 to 1 at 0.05 intervals. (b) Confusion matrix of each approach at the operating point. (c) Performance metrics of each approach. AUROC, area under the receiver operating characteristic c urve. The heatmaps were created with Python v3.8.8 (https://www.python.org) and Matplotlib v3.4.3 (https://www.matplotlib.org). The figure was generated using Inkscape v1.1 (https://inkscape.org).

	Kather et al.	Ours
Implementation	Matlab	Pytorch
Architecture	ResNet-18	[ResNet-18 v2, ShuffleNet v2 x1.0, EfficientNet-b0, ResNext-50]
Training	12.5% held-out validation set	5-fold cross validation
Early stopping tolerance	Three validation check	Six validation check
Validation check	256 iterations	256 iterations
Pretraining	ImageNet	ImageNet
Trainable layer	last 10 layers	[fully trainable, partial freeze]
Optimizer	Adam	Adam
L2-regularization	1e-4	1e-4
Leaning rate	1e-6	[1e-3, 1e-4, 1e-5, 1e-6]
Batch size	256	256
Max training duration	100 epochs	25,600 iteration
Augmentation	Geometric only	Geometric only
Confidence interval	500-fold bootstrapping	5 models from cross-validation

Supplementary Table 1. Comparison of a previous method and the search space used in this study.

			No. of patches (No. of cases)	
Cohort	Train/test	Fold #	MSS	MSI
CRC	Train set	1	9,084 (45)	9,781 (8)
		2	9,495 (44)	9,187 (8)
		3	8,691 (44)	11,347 (8)
		4	11,208 (44)	7,750 (8)
		5	8,226 (44)	8,639 (7)
		1+2+3+4+5	46,704 (221)	46,704 (39)
	Test set	-	70,569 (74)	28,335 (26)
STAD	Train set	1	9,201 (30)	11,858 (7)
		2	10,510 (30)	9,988 (7)
		3	10,579 (30)	10,497 (7)
		4	9,456 (30)	7,884 (7)
		5	10,539 (30)	10,058 (7)
		1+2+3+4+5	50,285 (150)	50,285 (35)
	Test set	-	90,104 (74)	27,904 (25)

MSS, Microsatellite stable; MSI, Microsatellite unstable

Supplementary Table 2. MSI dataset configuration

Architecture	Batch size	Learning rate	Total tensors	Trainable tensors	Trainable parameters	AUROC (95% CI)
ResNet-18 v2	256	1e-3	62	62	11177538	0.6967 (0.6551-0.7382)
ResNet-18 v2	256	1e-4	62	62	11177538	0.7639 (0.7242-0.8037)
ResNet-18 v2	256	1e-5	62	62	11177538	0.7833 (0.7306-0.8359)
ResNet-18 v2	256	1e-6	62	62	11177538	0.7693 (0.7345-0.8041)
ResNet-18 v2	256	1e-3	62	17	8394754	0.7816 (0.7420-0.8212)
ResNet-18 v2	256	1e-4	62	17	8394754	0.7933 (0.7473-0.8394)
ResNet-18 v2	256	1e-5	62	17	8394754	0.7991 (0.7720-0.8261)
ResNet-18 v2	256	1e-6	62	17	8394754	0.7938 (0.7635-0.8240)
ShuffleNet v2 x1.0	256	1e-3	170	170	1255654	0.7544 (0.7039-0.8049)
ShuffleNet v2 x1.0	256	1e-4	170	170	1255654	0.7433 (0.7068-0.7798)
ShuffleNet v2 x1.0	256	1e-5	170	170	1255654	0.7323 (0.7015-0.7632)
ShuffleNet v2 x1.0	256	1e-6	170	170	1255654	0.6243 (0.5571-0.6916)
ShuffleNet v2 x1.0	256	1e-3	170	47	980586	0.7504 (0.6957-0.8051)
ShuffleNet v2 x1.0	256	1e-4	170	47	980586	0.7451 (0.6975-0.7927)
ShuffleNet v2 x1.0	256	1e-5	170	47	980586	0.7280 (0.6800-0.7759)
ShuffleNet v2 x1.0	256	1e-6	170	47	980586	0.6047 (0.4952-0.7141)
EfficientNet-b0	96	1e-3	213	213	4010110	0.7395 (0.6689-0.8101)
EfficientNet-b0	96	1e-4	213	213	4010110	0.7983 (0.7606-0.8361)
EfficientNet-b0	96	1e-5	213	213	4010110	0.7917 (0.7734-0.8100)
EfficientNet-b0	96	1e-6	213	213	4010110	0.7673 (0.7250-0.8095)
EfficientNet-b0	96	1e-3	213	41	1895698	0.7946 (0.7263-0.8629)
EfficientNet-b0	96	1e-4	213	41	1895698	0.8052 (0.7602-0.8501)
EfficientNet-b0	96	1e-5	213	41	1895698	**0.8065 (0.7758-0.8373)**
EfficientNet-b0	96	1e-6	213	41	1895698	0.7267 (0.6765-0.7770)
Resnext50	84	1e-3	161	161	22984002	0.6646 (0.5398-0.7893)
Resnext50	84	1e-4	161	161	22984002	0.7968 (0.7696-0.8240)
Resnext50	84	1e-5	161	161	22984002	0.7819 (0.7543-0.8096)
Resnext50	84	1e-6	161	161	22984002	0.7871 (0.7790-0.7953)
Resnext50	84	1e-3	161	32	14548994	0.7825 (0.7279-0.8372)
Resnext50	84	1e-4	161	32	14548994	0.7640 (0.6825-0.8455)
Resnext50	84	1e-5	161	32	14548994	0.7741 (0.7482-0.8000)
Resnext50	84	1e-6	161	32	14548994	0.7870 (0.7637-0.8103)

Supplementary Table 3. Optimization of the MSI classification model

Architecture	Batch size	Learning rate	Total tensors	Trainable tensors	Trainable parameter	Average Accuracy (95%CI)	Average balanced accuracy (95%CI)
EfficientNet-b0	96	1e-3	213	213	4010110	0.7434 (0.6721-0.8147)	0.7245 (0.6366-0.8124)
EfficientNet-b0	96	1e-4	213	213	4010110	0.9409 (0.9206-0.9613)	0.9296 (0.9167-0.9425)
EfficientNet-b0	96	1e-5	213	213	4010110	**0.9523 (0.9424-0.9623)**	**0.9314 (0.9160-0.9468)**
EfficientNet-b0	96	1e-6	213	213	4010110	0.9460 (0.9419-0.9501)	0.9202 (0.9121-0.9284)
EfficientNet-b0	96	1e-3	213	41	1895698	0.9283 (0.9188-0.9379)	0.8949 (0.8813-0.9086)
EfficientNet-b0	96	1e-4	213	41	1895698	0.9379 (0.9358-0.9400)	0.9084 (0.9029-0.9140)
EfficientNet-b0	96	1e-5	213	41	1895698	0.9380 (0.9359-0.9401)	0.9078 (0.9043-0.9113)
EfficientNet-b0	96	1e-6	213	41	1895698	0.9379 (0.9358-0.9400)	0.9084 (0.9029-0.9140)

Supplementary Table 4. Optimization of the tissue classification model

	F1-score	
	Test A	Test B
All	0.9019	0.7290
Benign	0.9213	0.9143
Malignant	0.8488	0.6927

Supplementary Table 5. Evaluation of the gland detection model on test sets of the GlaS dataset

Lambda	Learning rate	Feature fraction	AUROC (95% CI)
0	0.001	0.5	0.6679 (0.6219-0.7139)
0	0.001	0.8	0.6658 (0.6185-0.7131)
0	0.001	1	0.6633 (0.6292-0.6973)
0	0.01	0.5	0.6656 (0.6194-0.7118)
0	0.01	0.8	0.6677 (0.6219-0.7134)
0	0.01	1	0.6690 (0.6235-0.7146)
0	0.1	0.5	0.6501 (0.6095-0.6907)
0	0.1	0.8	0.6634 (0.6249-0.7020)
0	0.1	1	0.6659 (0.6275-0.7043)
0.1	0.001	0.5	0.6695 (0.6242-0.7149)
0.1	0.001	0.8	0.6634 (0.6241-0.7027)
0.1	0.001	1	0.6657 (0.6317-0.6996)
0.1	0.01	0.5	0.6674 (0.6220-0.7127)
0.1	0.01	0.8	0.6709 (0.6235-0.7183)
0.1	0.01	1	0.6748 (0.6251-0.7246)
0.1	0.1	0.5	0.6544 (0.6085-0.7002)
0.1	0.1	0.8	**0.6749 (0.6365-0.7134)**
0.1	0.1	1	0.6629 (0.6333-0.6925)
0.2	0.001	0.5	0.6662 (0.6245-0.7079)
0.2	0.001	0.8	0.6632 (0.6239-0.7025)
0.2	0.001	1	0.6544 (0.6180-0.6907)
0.2	0.01	0.5	0.6644 (0.6188-0.7101)
0.2	0.01	0.8	0.6699 (0.6242-0.7155)
0.2	0.01	1	0.6684 (0.6209-0.7159)
0.2	0.1	0.5	0.6570 (0.6161-0.6978)
0.2	0.1	0.8	0.6705 (0.6424-0.6985)
0.2	0.1	1	0.6599 (0.6197-0.7001)

Supplementary Table 6. Optimization of the low-level approach
